# Symptoms and sources of *Yersinia enterocolitica*-infection: a case-control study

**DOI:** 10.1186/1471-2334-10-122

**Published:** 2010-05-20

**Authors:** Elisa Huovinen, Leila M Sihvonen, Mikko J Virtanen, Kaisa Haukka, Anja Siitonen, Markku Kuusi

**Affiliations:** 1 Epidemiologic Surveillance and Response Unit, National Institute for Health and Welfare (THL), Helsinki, Finland; 2 Bacteriology Unit, National Institute for Health and Welfare (THL), Helsinki, Finland

## Abstract

**Background:**

*Yersinia enterocolitica *(YE) is the causative agent of yersiniosis. YE encompass strains of diverse pathogenicity: YE biotypes 1B and 2-5 are considered pathogenic, whereas biotype 1A is in general considered nonvirulent. Also YE-like species, which can sometimes be misidentified as YE, are considered nonvirulent.

**Methods:**

In order to study differences in clinical picture caused by different YE types and their possible sources a case-control study was conducted in 2006. In this case-control study, 295 case-patients with YE or YE-like finding and their 758 controls responded to the questionnaire about symptoms and possible sources of infection.

**Results:**

Strains of pathogenic YE bio/serotypes 3-4/O:3 or 2/O:9 were found in 18%, YE biotype 1A in 65% and YE -like strains of 17% of the patients. Patients infected with the strains of pathogenic YE bio/serotypes were younger and had fever more often than those with BT 1A who suffered more from vomiting. Symptoms of reactive arthritis were reported by 10% of pathogenic YE infections, 3% of YE BT 1A, and 0.3% of the controls. Eating or tasting raw or medium done pork was a significant risk factor for pathogenic YE bio/serotype infection (OR 6.6; 95% CI 1.7-24.9) as well as eating in a canteen (OR 3.5; 95% CI 1.6-7.9). Imported fruits and berries were associated with increased risk of YE BT 1A finding.

**Conclusions:**

The symptoms of the patients with YE BT 1A differed from yersiniosis caused by the classic pathogenic YE bio/serotypes. In addition, the patients with YE BT 1A had more protracted gastrointestinal disorders and unspecific complaints. Small children were overrepresented in classic pathogenic bio/serotypes while in BT 1A or YE-like species were not found among children younger than two years. This suggests the lacking virulence of the BT 1A strains. We can not, however, rule out the possibility that some strains of genetically heterogeneous group of BT 1A may cause an illness.

## Background

*Yersinia enterocolitica *(YE) is a zoonotic bacterial species that causes food-transmitted infections. The most common clinical manifestation of a YE infection is self-limited gastroenteritis, but extraintestinal manifestations and postinfectious sequelae such as reactive arthritis occur as well [1]. YE infections are usually sporadic, although outbreaks have been reported [2-5]. Small household infection clusters may be more common than recognized [6]. Pigs are a known reservoir of YE strains [1] and raw pork products serve as an important vehicle for infection [7]. For example in Norway, undercooked pork, sausage products and untreated water have been associated with YE infections [8]. In the United States, contaminated tofu [9] and pasteurized milk [2] were found to be vehicles for YE in outbreaks.

YE species is divided into six biotypes (1A, 1B, 2, 3, 4, 5), which include several serotypes. The species encompasses both pathogenic strains including bio/serotypes 4/O:3 and 2/O:9 and strains which are classically considered non-pathogenic, namely strains of biotype (BT) 1A or non-biotypable strains. Closely related YE-like species, *Y. aldovae, Y. bercovieri, Y. frederiksenii, Y. intermedia, Y. kristensenii, Y. mollaretii, Y. rohdei, Y. alecksiciae, and Y. massiliensis*, can often be misidentified as YE [10]. YE-like species are also considered non-pathogenic[1]. However, several studies that suggest potential pathogenicity of BT 1A have been published [11-15].

In Finland, YE findings are notifiable and the National Institute for Health and Welfare (THL), formerly the National Public Health Institute (KTL), maintains the National Infectious Disease Register (NIDR) to which clinical microbiology laboratories report both culture- and antibody-confirmed yersinia findings. However, laboratories are not required to provide the bio/serotype information of the YE strains. In Finland, diarrhoeic stool samples are routinely cultured for presence of *Yersinia *strains in addition to *Salmonella*, *Campylobacter *and *Shigella*. Approximately 500 YE cases are reported yearly. Most of the YE infections are sporadic and only a few verified outbreaks have occurred in Finland. The most commonly notified pathogenic YE bio/serotypes are 4/O:3 and 2/O:9. However, the majority of all the notified findings consist of BT 1A YE and YE-like strains [10]. Correct identification of the strains that cause clinical symptoms is important for collecting reliable statistics to benefit decision-making in health care and food handling sectors. Therefore, we previously reported a laboratory study where methods to identify all YE isolates accurately where described [10]. The current case-control study focuses on symptoms and risk factors of the YE infections. The aims are to determine the clinical features caused by different YE bio/serotypes, especially to compare the traditionally pathogenic YE bio/serotypes to YE BT 1A and to identify the sources of infection in Finland.

## Methods

### Study design

All *Yersinia *strains (excluding *Y. pseudotuberculosis*) isolated from 41,848 stool specimens during January 1st 2006 through December 31st 2006 in 10 clinical microbiology laboratories were collected. Annually these 10 out of 22 laboratories report approximately two-thirds of all Finnish *Yersinia *cases. Laboratories were selected from different geographical locations to represent the whole Finland. The isolates were forwarded to the Bacteriology Unit (TABA; formerly Enteric Bacteria Laboratory), THL for further investigations. Identification of the *Yersinia *strains were described in detail earlier [10]. The amount of yersinia in stool samples was estimated semi-quantitatively by detecting growth on four streaking areas of CIN primary culture plates. If colonies that morphologically resembled *Yersinia *strains were detected only on the 1^st ^streaking area or only after cold-enrichment, the amount of *Yersinia *bacteria in stool of a patient was estimated to be low. If there were colonies on the 2^nd^, 3^rd ^or 4^th ^streaking area, the amount was estimated to be high. Bio/serotypes 3-4/O:3 or 2/O:9 are here called pathogenic YE types. Two nonbiotypable YE strains were analysed together with BT 1A strains and they are all here collectively called BT 1A.

During the study period, 462 YE and YE-like strains isolated from routinely submitted stool samples were found. Of those isolates, 29 were excluded from the present study, because another yersinia isolate from the same patient had already been included in the study. In addition, 27 isolates were excluded because also another microbe from the same patient was reported to NIDR (*Campylobacter *in 10 patients, *Salmonella *in 7, virus in 4, *Shigella *and *Cryptosporidium *in one patient each) or there was another YE finding within one week to half a year previously (four patients). Thus, 406 patients were accepted into this study.

In a case-control study, for each case patient four age-matched (same month and year of birth, widened if controls not found), gender-matched and geographically-matched (same municipality of residence) controls were selected from the Finnish population register every two weeks. A detailed questionnaire was sent to each case immediately after their yersinia isolate was received by TASU. For the cases, questions on exposures covered the time period of two weeks before the onset of the symptoms (or if person had no symptoms, to the time period two weeks before the stool sample was given). For the controls, the time period covered two weeks before answering the questionnaire. A reminder with a new questionnaire form was sent two weeks later to the case or the control if there was no response.

The questionnaire included questions related to symptoms and sequelae of yersinia infection as well as to specific exposures including consumption of different food items and travelling abroad. Questionnaires were identical for the cases and controls, except the questions concerning the yersinia infection. Instead, controls were asked if they had had diarrhoea or abdominal cramps with fever during the previous month or if they had ever had yersinia infection. Joint symptoms were asked identically from cases and controls. A probable reactive arthritis in a case or control was defined as swelling with gleam/redness in a joint and the onset of joint symptoms reported with the accuracy of 1-3 days.

The study was approved by the Ethics Committee of THL.

### Statistical analysis

Comparisons of age, gender and symptoms between patients with different bio/serotypes were made using either two-sample t-tests (age and duration of symptoms) or chi square test (others). Source analyses were done using univariate conditional logistic regressions, *i.e. *between cases and controls, separately for pathogenic YE and YE BT 1A. For each source, missing values (due to missing choice, "unknown" or "do not remember") were omitted from the analysis. Cases were included in the source analyses if the onset of symptoms was reported with the accuracy of 1-3 days and the time from the onset of symptoms to the stool sample was not longer than six weeks. Controls were included in the analyses if their case was included. All analyses were made using Stata version 9.2.

## Results

### Defining the patients to the study

Of the 406 patients, pathogenic YE were found in stool samples of 74 (18%), BT 1A strains of 263 (65%) and YE -like strains of 69 (17%) subjects. YE-like strains included *Y. bercovieri, Y. frederiksenii, Y. intermedia, Y. kristensenii, Y. mollaretii *and *Y. rohdei *(table 1). Patients with pathogenic YE types were younger than patients with other YE types. Especially small children under two years of age were overrepresented among patients with pathogenic YE (16%, 12/74), while none of the BT 1A or YE-like species were found among children younger than two years.

**Table 1:** *Yersinia *strains isolated from 406 patients by bio/serotype and demographical characteristics of the patients, Finland, 2006.

	*Number of strains (%)*	*Mean age, years (min max)*	*Proportion of men N (%)*
***Y. enterocolitica***			
3-4/O:3 or 2/O:9	74 (18%)	32 (0.6-80)	41 (55%)
1A or non-biotypable	263 (65%)	50 (2-99) *	98 (37%) *
***Y. enterocolitica- *like species**			
*Y. bercovieri*	17 (4%)	44 (5-81) *	3 (18%) *
*Y. frederiksenii*	26 (6%)	42 (3-73)	10 (38%)
*Y. intermedia*	8 (2%)	68 (40-87) *	5 (63%)
*Y. kristensenii*	4 (1%)	48 (23-79)	2 (50%)
*Y. mollaretii*	12 (3%)	62 (26-93) *	2 (17%) *
*Y. rohdei*	2 (0.5%)	55 (47-63)	1 (50%)

* p < 0.05 for the difference compared to YE bio/serotypes 3-4/O:3 or 2/O:9

For the case-control study, questionnaires were sent to the 406 case patients and for their 1597 controls (for some patients it was not possible to find four controls). In total, 301 of 406 cases (74%) and 1002 of 1597 controls (62%) returned the questionnaire. Only those strata with at least one case and control were useable. Thus 295 cases (73%) and 758 controls (47%) were included. On average each case had 2.6/4 controls (64%). Accepted cases were younger (p = 0.04), while no significant difference was found between men and women (p = 0.08).

Among 295 cases included in analysis of the case-control study, 61 (21%) had a strain of pathogenic YE type, 187 (63%) had YE BT 1A and 47 (16%) had a YE-like strain. Since YE-like species of the cases comprised of six different species, the numbers of the cases with any one species were too small for further analyses. Therefore, the analyses were done only among cases with pathogenic (bio/serotypes 3-4/O:3 or 2/O:9) or YE BT 1A (BT 1A or non-biotypable) and their controls.

### Symptoms

Almost all cases (240/248) had at least one of the five symptoms asked (i.e. diarrhoea, fever, stomach cramps, vomiting or blood in stools). One of the 61 cases (2%) with pathogenic YE and 7 of 187 cases (4%) with BT 1A reported having had none of these symptoms. Time from the onset of the symptoms to the stool sample varied widely among patients with BT 1A and 40% of them were unable to define the accurate onset of symptoms (Table 2).

**Table 2:** The time from onset of symptoms to stool sample taking.

*Time from the onset of symptoms to sample taking*	*Bio/serotype 3-4/O:3 or 2/O:9*	*Biotype 1A*	***p-value***^***1***^
**0 - 2 weeks**	61% (37/61)	36% (67/187)	
**2 - 4 weeks**	20% (12/61)	11% (20/187)	
**4 - 6 weeks**	8% (5/61)	6% (11/187)	
**Over 6 weeks**	0	8% (15/187)	
**Not known**^**2**^	11% (7/61)	40% (74/187)	
**mean:(min- max) **^**3**^	12 days:(1-39)	25 days:(0 -360)	P = 0.03

**^1 ^**P for the difference between YE bio/serotypes 3-4/O:3 or 2/O:9 and biotype 1A

^2 ^No date or inexact date for the onset of symptoms or sample taken before symptoms began

^3^Subjects with not known onset of symptoms excluded

**Figure 1:**
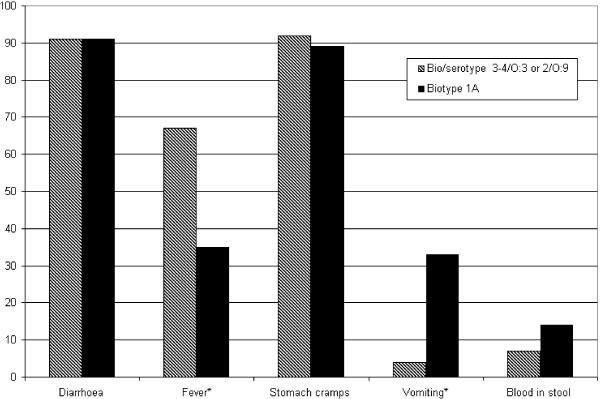
**Symptoms of the patients**. Symptoms among the subjects, from whom YE were isolated at the latest six weeks from the beginning of symptoms. Subjects with missing or unclear answers were excluded. *Statistically significant difference between bio/serotypes 3-4/O:3 or 2/O:9 and BT1A (P < 0.001).

To decrease the recall bias we analysed only the symptoms of the cases with different bio/serotypes among those who were able to report the accurate onset of symptoms (1-3 days) and whose sample was taken within six weeks from onset of symptoms. With these criteria 54/61 (89%) cases with pathogenic YE and 98/187 (52%) cases with YE BT1A finding were included to the symptom analyses. Diarrhoea (91%) and stomach cramps (89-92%) were the most common symptoms, with no difference between the cases with pathogenic YE or BT 1A (Figure 1). Fever was more common among cases with pathogenic YE than among those with BT 1A (67% and 35% respectively P < 0.001). One third of the cases with BT 1A reported vomiting while among those with pathogenic type vomiting was rare (33% and 4% respectively P < 0.001). The amount of bacteria was high in 66% (38/58) of stool samples with pathogenic YE while with BT 1A high amounts were found only in 19% (35/184) of stool samples. However, within the different bio/serotypes the occurrence of symptoms was not associated with high amount of bacteria detected in stool samples (data not shown).

Of the 758 controls responding to the questionnaire, 127 (17%) reported having had diarrhoea and 16 (2%) having had abdominal cramps with fever during the previous month. Nine controls (1%) reported of previous yersinia infection. No other intestinal bacteria were reported to NIDR for controls during the previous five months.

38% of the cases with pathogenic YE and 45% of the cases with BT 1A reported having had at least one joint symptom (ache, pain associated to motion, swelling or gleam/redness). Also 30% of the controls reported at least one joint symptom. When only those reporting the onset of joint symptoms at least with the accuracy of 1-3 days were considered, the prevalence of joint symptoms was lower, especially among controls (4%). Among cases with pathogenic YE this proportion was 28% and among cases with BT 1A it was 20%. Six cases with pathogenic YE type (10%), five cases with BT 1A (3%) and two controls (0.3%) had probable reactive arthritis.

Self-reported gastro-intestinal diseases were common among cases with BT 1A (24%), compared to only 10% among cases with pathogenic YE (7% and 6% among their controls respectively). Continuous diarrhoea was common among cases with BT 1A (20%), while among cases with pathogenic YE and all controls it was rare (2-3%). Special diets were more common among cases with BT 1A than among their controls, especially they used more often diet low in lactose (25%) compared to their controls (15%).

### Sources

Cases with the defined onset of symptoms (54 cases with pathogenic YE type and 98 with BT 1A) and their controls (133 and 251 respectively) were included in the risk factor analyses. For each source, missing, unknown and 'do not remember' answers were omitted from the analysis.

Eating or tasting raw or medium done pork was a significant risk factor for pathogenic YE infection (OR 6.6: 95% CI 1.7-24.9). Non-pasteurized milk products increased the risk of infection but this effect was not statistically significant. Eating outside home was associated with the pathogenic YE infection, both eating in a restaurant or in a canteen-type place increased the risk (Table 3). Travelling abroad was associated with the pathogenic YE infections. Forty-one percent (22/54) of the patients with the pathogenic YE strain reported having travelled abroad during the two weeks preceding the onset of symptoms, but only 8% (10/133) of their controls. Of the patients with the pathogenic YE who had travelled abroad, 23% (5/22) had travelled outside Europe, compared to 11% (1/9) of their controls.

**Table 3:** Risk factors of YE bio/serotype 3-4/O:3 or 2/O:9 and biotype 1A finding, Finland, 2006.

	3-4/O:3 or 2/O:9		Biotype 1A	
**Number of cases and controls**^**1**^	54 cases 133 controls		98 cases 251 controls	
	**exposed/all**^**1**^ **-cases** **-controls**	**OR (95%CI) **^**2**^	**exposed/all**^**1**^ **-cases** **-controls**	**OR (95%CI) **^**2**^
**Traveling**				
-abroad	22/54 10/133	39.4(5.2-296.7)*	36/97 21/248	8.0(3.8-16.9)*
-in Finland	8/54 38/133	0.5(0.2-1.0)	21/97 78/246	0.6(0.3-1.0)
**Eating outside home**				
-in a restaurant, coffee house etc. ^**3**^	48/53 95/130	6.1(1.4-27.2)*	72/93 164/242	1.8(0.9-3.7)
-in a staff , school or daycare canteen	22/48 30/127	3.5(1.6-7.9)*	36/87 78/232	1.5(0.8-2.7)
**Meat and meat products**:				
-pork (well done)	46/51 119/132	1.2(0.4-3.4)	84/93 222/239	0.7(0.3-1.6)
-pork (medium or raw)	10/41 4/108	6.6(1.7-24.9)*	3/66 14/166	0.2(0.0-1.6)
-bovine (well done)	42/51 110/132	1.0(0.4-2.5)	77/90 206/233	0.8(0.4-1.7)
-bovine (medium or raw)	10/40 18/110	1.6(0.6-4.3)	14/64 38/173	1.0(0.5-2.2)
-salami	23/51 66/126	0.8(0.4-1.5)	38/86 119/222	0.7(0.4-1.2)
-poultry	48/51 111/126	3.1(0.7-13.8)	81/90 207/240	1.3(0.6-2.9)
-sheep	6/45 17/117	1.6(0.5-5.0)	7/81 22/203	0.8(0.3-2.0)
-game	9/49 25/124	0.8(0.3-2.0)	14/86 65/226	0.5(0.2-0.9)*
**Milk and milk products**				
-pasteurized	47/52 124/129	0.3(0.1-1.5)	88/97 241/249	0.4(0.1-1.0)*
-unpasteurized	10/46 13/123	2.4(0.9-6.2)	8/85 30/218	0.6(0.2-1.3)
**Soybean and soybean products**	6/49 22/126	0.6(0.2-1.6)	15/87 37/227	1.2(0.6-2.4)
**Fruits and berries**				
-domestic	37/52 105/128	0.6(0.3-1.3)	75/89 221/243	0.6(0.3-1.3)
-imported	46/53 112/127	0.9(0.3-2.4)	91/95 210/239	3.5(1.2-10.5)*
**Vegetables**				
-roots^**4**^	35/49 100/129	0.9(0.4-2.0)	77/89 200/237	1.4(0.7-2.9)
-lettuce and cabbage^**5**^	44/52 112/129	1.0(0.4-3.0)	84/96 229/240	0.3(0.1-0.8)*
-onion and leek	35/50 98/130	0.8(0.4-1.9)	63/91 193/237	0.5(0.3-1.0)*
-cucumber, tomato, pepper	49/53 127/130	0.2(0.0-1.5)	94/96 245/247	0.4(0.0-5.1)
**Washing vegetables **^**6**^				
-washing roots	38/39 112/116	0.9(0.1-9.8)	69/72 217/224	0.2(0.0-1.4)
-washing pre-washed roots	34/35 102/108	1.6(0.2-14.3)	60/68 196/206	0.2(0.0-0.6)*
-pealing roots	36/39 98/113	2.2(0.6-8.2)	66/73 185/216	1.3(0.5-3.3)
-washing vegetables other than roots	42/43 112/119	2.7(0.3-23.0)	69/75 223/231	0.1(0.0-0.7)*
-washing pre-washed vegetables other than roots	33/36 101/111	1.3(0.3-5.1)	51/62 196/206	0.1(0.0-0.4)*

^**1**^Cases were included to the source analyses if the onset of symptoms was reported with the accuracy of a couple of days and time from the onset of symptoms to the stool sample was not longer than six weeks. Controls were included if their case was included. For each source group, missing values were omitted per analysis.

^**2**^Only those strata with at least case and one control having non-missing values were included to logistic regression analyses. These omissions were done separately per source.

^**3**^Restaurant, coffee house, hamburger restaurant, chip shop, pizzeria, service station restaurant, snack bar

^**4**^Carrot, Swedish turnip, radish

^**5**^White cabbage, red cabbage, cauliflower, lettuce, iceberg lettuce, Chinese cabbage

^**6**^exposed are those who washed vegetables usually or always, compared to those washing them only rarely

* Statistically significant result

Imported fruits and berries were the only food associated with increased risk of YE BT 1A finding, while eating game, pasteurized milk and milk products, some vegetables and domestic berries decreased the risk. Those who rarely washed vegetables were in increased risk of YE BT 1A. Eating in restaurant (OR 2.8: 95% CI 1.6-4.9) and in cafeteria (1.8: 1.0-3.0) increased risk of BT 1A finding. Traveling abroad was more common among patients with BT 1A (37%, 36/97) than in their controls (8%, 21/248). Countries outside Europe were a common destination, in 49% (17/35) of journeys of patients with BT 1A compared to 15% (3/21) of their controls.

## Discussion

Majority (65%) of the culture-confirmed YE findings in the present study were of BT 1A. Bio/serotypes 3-4/O:3 or 2/O:9 were found in 18% of the patients. This agrees with BT 1A prevalence found in Switzerland and Great Britain, where majority (60% and 53%, respectively) of the human YE findings were of BT 1A [12,16]. The role of YE BT 1A in causing human disease has been debated [11-15,17-19]. The mechanisms behind the potential pathogenicity of this biotype have remained unclear, since YE BT 1A usually lack the classical virulence markers of *Yersinia *virulence plasmid pYV and functional chromosomal virulence genes [19].

Majority of the patients with pathogenic YE had diarrhoea, stomach cramps and fever, while vomiting was rare. This agrees with the typical clinical picture of yersiniosis. The symptoms of patients with YE BT 1A differed from those of pathogenic biotypes by having less often fever and more often vomiting. This is in contrast with a study where illness experienced by patients with BT 1A was indistinguishable from that caused by pathogenic YE [12]. The higher frequency of vomiting among patients with YE BT 1A might be due to enterotoxin YstB known to be produced by some BT 1A strains [20]. Alternatively, vomiting among patients with YE BT 1A, may suggest that the symptoms were due to some other reason, potentially to viral infection and YE was found just by chance. Viral enteritis is a common disease but in protracted gastrointestinal diseases, stool samples are usually investigated for bacterial enteropathogens and for certain parasites, but viruses are rarely searched for.

Time from the onset of symptoms to the stool sample varied a lot among patients with YE BT 1A, whereas among patients with pathogenic YE it was less than two weeks in majority of the cases, the longest reported time being 39 days. About half of the patients, irrespective of the YE bio/serotype found, informed that the stool sample was taken due to protracted gastrointestinal symptoms. This may be a sign of a persistence of the YE infections. In a Norwegian study it was found that both adults and children carried YE in the stool for extended periods [21]. The patients with YE BT 1A strain had more prolonged gastrointestinal disorders than their controls. These findings suggest that some BT 1A strains may have an adverse effect on gastrointestinal tract and possibly worsen chronic gastrointestinal symptoms or the patient's immune system may already be compromised. This agrees with an observation that patients with non-steroid anti-inflammatory drug-induced colitis had a relatively high prevalence of YE [22]. Genetic differences among YE BT 1A strains that may be linked to their virulence have also been reported [12,23,24]. Also we have detected several genetically different subgroups among the YE BT 1A strains included in the present study (unpublished results).

The number of reported joint symptoms in our study was high among both cases and controls. A high number of joint symptoms among controls have been detected in other population-based studies earlier [25]. However, when we limited the typical symptoms of reactive arthritis, swelling with gleam/redness with being able to report the accurate onset of symptoms were found these symptoms with 10% pathogenic YE bio/serotypes, 3% YE BT 1A and 0.3% of controls. Still, it is possible that symptoms of reactive arthritis may have appeared to some patients only after they had already answered the questionnaire.

Small children were overrepresented among patients with pathogenic YE and only pathogenic YE strains were found in children younger than two years. The association of pathogenic YE with small children have been shown in earlier studies [26-28]. Whether there are specific risk factors for small children or whether their immature immune system increases the risk of infection could not be defined in this study. A Swedish study found the use of baby's dummy, contact with domestic animals, eating treated sausage or food prepared from raw pork products as risk factors for YE infection in children [29]. However, the YE BT 1A seems not to be associated with small children. Since children are known to be more susceptible to enteric infections due to immature immune system [30], this observation implies that YE BT 1A unlikely causes a disease in otherwise healthy persons.

The results of the study show that the sources for pathogenic YE and YE BT 1A were different. Eating or tasting raw or medium done pork was significantly associated with finding of pathogenic YE bio/serotypes while no association with finding of YE BT 1A was found. Pork has also been the major source of pathogenic YE infections in earlier studies [1,7,31]. Sources of YE BT 1A has been shown to be very diverse *e.g. *water, vegetables, and different animals [16,19,32]. Pasteurized milk has been associated with YE infections [2], but we found an association with non-pasteurized milk products. In our study pasteurized milk products were used less by patients with BT 1A than by their controls.

Analyses on possible sources were restricted due to the small number of pathogenic YE strains in our study. A problem in tracing the sources in a questionnaire study was caused by the time delay from the infection to answering of the questionnaire. Patients were enquired of the possible sources of their YE infection in a two-week period before the onset of symptoms. For the controls, on the other hand, the questions concerned the time period two weeks before answering the questionnaire. The time from the onset of symptoms to responding the questionnaire varied a lot among patients and many could not report the exact time of the onset of symptoms. To control these differences and to minimize recall bias we included in the analysis only cases who reported the onset of symptoms accurately and who have had at most six weeks from the onset of symptoms to sample taking.

## Conclusions

The majority of YE strains isolated from Finnish patients belonged to BT 1A. The symptoms and sources of patients with YE BT 1A were different from the pathogenic YE. In addition, BT 1A patients had more unspecific complaints and protracted gastrointestinal disorders, which suggest that the original cause of the illness may have been other than YE BT 1A. This is supported by the fact, that we did not find any YE BT 1A strains in small children. However, it is still possible that in the highly heterogeneous group of YE BT 1A there are subgroups that can cause a disease, but this needs further investigations. While the significance of the YE BT 1A strains remains unclear, the correct registering of infections caused by the truly pathogenic YE strains is essential for health protection purposes.

## Competing interests

The authors declare that they have no competing interests.

## Authors' contributions

EH participated in the design and analyses of the study and drafted the manuscript. LMS participated in design of the study, collecting the bacterial strains and their identification, and drafting the manuscript. MJV participated in analyses of the case-control study. KH participated in the design of the study, especially the microbiology lab works, and drafting the manuscript. AS participated in the design of the study and analysis of the microbiological results. MK participated in the design of the study and analyses of the case-control study. All authors read and approved the final manuscript.

## Pre-publication history

The pre-publication history for this paper can be accessed here:

http://www.biomedcentral.com/1471-2334/10/122/prepub
